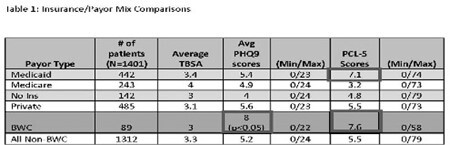# 569 Insurance Payor Increases the Incidence of Depression and PTSD in Burn Patients

**DOI:** 10.1093/jbcr/irae036.203

**Published:** 2024-04-17

**Authors:** Laura K Pezzopane, John Loftus, Beth McGuire, Toni Bean, Nicole Fornalski, Nicole P Bernal

**Affiliations:** The Ohio State Wexner Medical Center, Columbus, OH; The Ohio State University Wexner Medical Center, Columbus, OH; Ohio State Medical Center, Columbus, OH; The Ohio State Wexner Medical Center, Columbus, OH; The Ohio State University Wexner Medical Center, Columbus, OH; Ohio State Medical Center, Columbus, OH; The Ohio State Wexner Medical Center, Columbus, OH; The Ohio State University Wexner Medical Center, Columbus, OH; Ohio State Medical Center, Columbus, OH; The Ohio State Wexner Medical Center, Columbus, OH; The Ohio State University Wexner Medical Center, Columbus, OH; Ohio State Medical Center, Columbus, OH; The Ohio State Wexner Medical Center, Columbus, OH; The Ohio State University Wexner Medical Center, Columbus, OH; Ohio State Medical Center, Columbus, OH; The Ohio State Wexner Medical Center, Columbus, OH; The Ohio State University Wexner Medical Center, Columbus, OH; Ohio State Medical Center, Columbus, OH

## Abstract

**Introduction:**

Intriduction: Work-related burn injuries have the added stress of dependence on a third-party payor. Patients using Bureau of Worker’s Compensation (BWC) report frustration and dissatisfaction with the claim coverage process. In 2021, based on our initial study's result, we began using the Patient Health Questionnaire-9 (PHQ-9), a 9-item DSM-IV-based self-reported depression screening tool. We added the PTSD Checklist (PCL-5), a 20-question self-administered screening tool to screen for Post-Traumatic Stress Disorder (PSTD). We hypothesized that there is a correlation between insurance type and increased scores on the PHQ-9 and PCL-5. We hypothesized that the risk of depression and PTSD is affected by a patient’s access to care, referral approval, and financial burden of treatment, and differs based on payor.

**Methods:**

Method: A retrospective review of our outpatient database was conducted of burn clinic visits with a completed PHQ-9 and PCL-5 between 7/1/2021-6/30/2023. This was considered exempt by the IRB (Institutional Review Board) committee based on use of deidentified database. The PHQ-9 is completed at initial visit and then once every 2 weeks; the PCL-5 on initial visit and then once per month. This data was recorded in the EMR (Electronic Medical Record) flowsheet and then transferred to the deidentified database. Additional information analyzed included total body surface area (TBSA) and need for admission and/or surgical procedure.

**Results: Results:**

1401 visits with PHQ-9 and PCL-5 collection were reviewed. The average scores for both the PHQ-9 and PCL-5 were higher with patients that had BWC as their payor with a significant difference in the PHQ-9. (Table 1) The TBSA was significantly higher in the patients that underwent surgery and/or admission. This group of patients, those that required surgery had significantly higher PHQ-9, and a trend toward higher PCL-5 scores. (Table 2) When divided into the payors, the BWC group has the highest PHQ-9 and PCL-5 scores, but total patients are too small in each group to establish significance.

**Conclusions: Conclusions:**

Compared to the preliminary study, we can say with confidence that patients with BWC are at higher risk for depression and PTSD. The limitation of this study design does not allow us to determine causality, and patient reported outcomes would be necessary. There is an additional increased risk of depression and PTSD if the outpatient requires surgery with or without admission.

**Applicability of Research to Practice: Applicability of research to practice:**

This research is designed to identify specific needs in work-related injuries in hopes of BWC changing this practice.